# Supplementary material for: OpenMM 7: Rapid development of high performance algorithms for molecular dynamics
Source: PLoS Comput Biol. 2017 Jul 26;13(7):e1005659. doi: 10.1371/journal.pcbi.1005659 (PMC5549999; doi:10.1371/journal.pcbi.1005659)
Supplement: S1 Source Code — (ZIP) [file pcbi.1005659.s002.zip › OpenMM-7.0.1-Source/docs/api-python/generated/simtk.openmm.openmm.MonteCarloMembraneBarostat.html]

MonteCarloMembraneBarostat — OpenMM 7.0.1.dev-5e86c4f documentation


# MonteCarloMembraneBarostat¶

*class* `simtk.openmm.openmm.``MonteCarloMembraneBarostat`(*\*args*)¶
:   This is a Monte Carlo barostat designed specifically for membrane simulations. It assumes the membrane lies in the XY plane. The Monte Carlo acceptance criterion includes a term to model isotropic pressure, which depends on the volume of the periodic box, and a second term to model surface tension, which depends on the cross sectional area of the box in the XY plane. Note that pressure and surface tension are defined with opposite senses: a larger pressure tends to make the box smaller, but a larger surface tension tends to make the box larger.

    There are options for configuring exactly how the various box dimensions are allowed to change:

    > - The X and Y axes may be treated isotropically, in which case they always scale by the same amount and remain in proportion to each other; or they may be treated anisotropically, in which case they can vary independently of each other.
    > - The Z axis can be allowed to vary independently of the other axes; or held fixed; or constrained to vary in inverse proportion to the other two axes, so that the total box volume remains fixed.

    This class assumes the simulation is also being run at constant temperature, and requires you to specify the system temperature (since it affects the acceptance probability for Monte Carlo moves). It does not actually perform temperature regulation, however. You must use another mechanism along with it to maintain the temperature, such as LangevinIntegrator or AndersenThermostat.

    `__init__`(*self*, *defaultPressure*, *defaultSurfaceTension*, *temperature*, *xymode*, *zmode*, *frequency=25*) → MonteCarloMembraneBarostat¶
    :   \_\_init\_\_(self, defaultPressure, defaultSurfaceTension, temperature, xymode, zmode) -> MonteCarloMembraneBarostat
        \_\_init\_\_(self, other) -> MonteCarloMembraneBarostat

        Create a MonteCarloMembraneBarostat.

        |  |  |
        | --- | --- |
        | Parameters: | - **defaultPressure** (*double*) – the default pressure acting on the system (in bar) - **defaultSurfaceTension** (*double*) – the default surface tension acting on the system (in bar\*nm) - **temperature** (*double*) – the temperature at which the system is being maintained (in Kelvin) - **xymode** (*XYMode*) – the mode specifying the behavior of the X and Y axes - **zmode** (*ZMode*) – the mode specifying the behavior of the Z axis - **frequency** (*int*) – the frequency at which Monte Carlo volume changes should be attempted (in time steps) |

    Methods

    |  |  |
    | --- | --- |
    | `Pressure`(() -> std::string const &) | This is the name of the parameter which stores the current pressure acting on the system (in bar). |
    | `SurfaceTension`(() -> std::string const &) | This is the name of the parameter which stores the current surface tension acting on the system (in bar\*nm). |
    | `__init__`((self, defaultPressure, ...[, ...]) | \_\_init\_\_(self, defaultPressure, defaultSurfaceTension, temperature, xymode, zmode) -> MonteCarloMembraneBarostat |
    | `getDefaultPressure`((self) -> double) | Get the default pressure acting on the system (in bar). |
    | `getDefaultSurfaceTension`((self) -> double) | Get the default surface tension acting on the system (in bar\*nm). |
    | `getForceGroup`((self) -> int) | Get the force group this Force belongs to. |
    | `getFrequency`((self) -> int) | Get the frequency (in time steps) at which Monte Carlo volume changes should be attempted. |
    | `getRandomNumberSeed`((self) -> int) | Get the random number seed. |
    | `getTemperature`((self) -> double) | Get the temperature at which the system is being maintained, measured in Kelvin. |
    | `getXYMode`(...) | Get the mode specifying the behavior of the X and Y axes. |
    | `getZMode`(...) | Get the mode specifying the behavior of the Z axis. |
    | `setDefaultPressure`(self, pressure) | Set the default pressure acting on the system. |
    | `setDefaultSurfaceTension`(self, surfaceTension) | Set the default surface tension acting on the system. |
    | `setForceGroup`(self, group) | Set the force group this Force belongs to. |
    | `setFrequency`(self, freq) | Set the frequency (in time steps) at which Monte Carlo volume changes should be attempted. |
    | `setRandomNumberSeed`(self, seed) | Set the random number seed. |
    | `setTemperature`(self, temp) | Set the temperature at which the system is being maintained. |
    | `setXYMode`(self, mode) | Set the mode specifying the behavior of the X and Y axes. |
    | `setZMode`(self, mode) | Set the mode specifying the behavior of the Z axis. |
    | `usesPeriodicBoundaryConditions`((self) -> bool) | Returns whether or not this force makes use of periodic boundary conditions. |

    Attributes

    |  |  |
    | --- | --- |
    | `ConstantVolume` |  |
    | `XYAnisotropic` |  |
    | `XYIsotropic` |  |
    | `ZFixed` |  |
    | `ZFree` |  |

    *static* `Pressure`() → std::string const &¶
    :   This is the name of the parameter which stores the current pressure acting on the system (in bar).

    *static* `SurfaceTension`() → std::string const &¶
    :   This is the name of the parameter which stores the current surface tension acting on the system (in bar\*nm).

    `getDefaultPressure`(*self*) → double¶
    :   Get the default pressure acting on the system (in bar).

        |  |  |
        | --- | --- |
        | Returns: | the default pressure acting on the system, measured in bar. |
        | Return type: | double |

    `setDefaultPressure`(*self*, *pressure*)¶
    :   Set the default pressure acting on the system. This will affect any new Contexts you create, but not ones that already exist.

        |  |  |
        | --- | --- |
        | Parameters: | **pressure** (*double*) – the default pressure acting on the system, measured in bar. |

    `getDefaultSurfaceTension`(*self*) → double¶
    :   Get the default surface tension acting on the system (in bar\*nm).

        |  |  |
        | --- | --- |
        | Returns: | the default surface tension acting on the system, measured in bar\*nm. |
        | Return type: | double |

    `setDefaultSurfaceTension`(*self*, *surfaceTension*)¶
    :   Set the default surface tension acting on the system. This will affect any new Contexts you create, but not ones that already exist.

        |  |  |
        | --- | --- |
        | Parameters: | **surfaceTension** (*double*) – the default surface tension acting on the system, measured in bar. |

    `getFrequency`(*self*) → int¶
    :   Get the frequency (in time steps) at which Monte Carlo volume changes should be attempted. If this is set to 0, the barostat is disabled.

    `setFrequency`(*self*, *freq*)¶
    :   Set the frequency (in time steps) at which Monte Carlo volume changes should be attempted. If this is set to 0, the barostat is disabled.

    `getTemperature`(*self*) → double¶
    :   Get the temperature at which the system is being maintained, measured in Kelvin.

    `setTemperature`(*self*, *temp*)¶
    :   Set the temperature at which the system is being maintained.

        |  |  |
        | --- | --- |
        | Parameters: | **temp** (*double*) – the system temperature, measured in Kelvin. |

    `getXYMode`(*self*) → OpenMM::MonteCarloMembraneBarostat::XYMode¶
    :   Get the mode specifying the behavior of the X and Y axes.

    `setXYMode`(*self*, *mode*)¶
    :   Set the mode specifying the behavior of the X and Y axes.

    `getZMode`(*self*) → OpenMM::MonteCarloMembraneBarostat::ZMode¶
    :   Get the mode specifying the behavior of the Z axis.

    `setZMode`(*self*, *mode*)¶
    :   Set the mode specifying the behavior of the Z axis.

    `getRandomNumberSeed`(*self*) → int¶
    :   Get the random number seed. See setRandomNumberSeed() for details.

    `setRandomNumberSeed`(*self*, *seed*)¶
    :   Set the random number seed. It is guaranteed that if two simulations are run with different random number seeds, the sequence of Monte Carlo steps will be different. On the other hand, no guarantees are made about the behavior of simulations that use the same seed. In particular, Platforms are permitted to use non-deterministic algorithms which produce different results on successive runs, even if those runs were initialized identically.

        If seed is set to 0 (which is the default value assigned), a unique seed is chosen when a Context is created from this Force. This is done to ensure that each Context receives unique random seeds without you needing to set them explicitly.

    `usesPeriodicBoundaryConditions`(*self*) → bool¶
    :   Returns whether or not this force makes use of periodic boundary conditions.

        |  |  |
        | --- | --- |
        | Returns: | true if force uses PBC and false otherwise |
        | Return type: | bool |

    `__delattr__`¶
    :   x.\_\_delattr\_\_(‘name’) <==> del x.name

    `__format__`()¶
    :   default object formatter

    `__getattribute__`¶
    :   x.\_\_getattribute\_\_(‘name’) <==> x.name

    `__hash__`¶

    `__reduce__`()¶
    :   helper for pickle

    `__reduce_ex__`()¶
    :   helper for pickle

    `__sizeof__`() → int¶
    :   size of object in memory, in bytes

    `__str__`¶

    `getForceGroup`(*self*) → int¶
    :   Get the force group this Force belongs to.

    `setForceGroup`(*self*, *group*)¶
    :   Set the force group this Force belongs to.

        |  |  |
        | --- | --- |
        | Parameters: | **group** (*int*) – the group index. Legal values are between 0 and 31 (inclusive). |

High performance molecular simulation on GPUs


### Navigation

- Application Layer
- Library Layer
  - Core Objects
  - Forces
  - Integrators
  - Extras

©2015, Stanford University and the Authors.
|
Powered by Sphinx 1.3.1
& Alabaster 0.7.7
|
Page source
